# The mediating role of out-of-hospital adherence between health literacy and quality of life in Chinese stroke survivors: a cross-sectional study

**DOI:** 10.3389/fpubh.2026.1917398

**Published:** 2026-10-07

**Authors:** Hui Jun Sun, Jia Lu Cao, Jia Yan Qu, Mei Ting Wu, Jian Ping Lu

**Affiliations:** 1 Shanghai Clinical Research and Trial Center, ShanghaiTech University, Shanghai, China; 2 Nursing Department, Shanghai First Maternity and Infant Hospital, Tongji University School of Medicine, Shanghai, China; 3 Department of Vascular Surgery, Yueyang Hospital of Integrated Traditional Chinese Medicine, Shanghai, China; 4 School of Nursing and Health Management, Shanghai University of Medicine & Health Sciences, Shanghai, China; 5 Nursing Department, Gushengtang Holdings Co., Ltd., Shanghai, China

**Keywords:** adherence, health literacy, healthy aging, older adults, public health intervention, quality of life, stroke

## Abstract

**Background:**

Stroke, the second foremost cause of mortality globally, imposes a considerable disease burden in China. Limited health literacy and difficulties adhering to post-discharge care may be associated with poorer quality of life among stroke survivors. However, the extent to which out-of-hospital adherence statistically accounts for the association between health literacy and quality of life remains insufficiently characterized.

**Objective:**

To examine associations among health literacy, out-of-hospital adherence, and quality of life, and to assess whether out-of-hospital adherence statistically mediates the association between health literacy and quality of life in a cross-sectional sample of stroke survivors.

**Methods:**

A cross-sectional study included 509 participants recruited through convenience sampling from four traditional Chinese medicine hospitals in Shanghai. Health literacy, out-of-hospital adherence, and stroke-specific quality of life were assessed using Chinese-language instruments. Statistical mediation was examined using PROCESS Model 4 with 5,000 bootstrap resamples.

**Results:**

Stroke survivors’ health literacy averaged 31.40 ± 6.78 (below threshold), with analysis skills scoring lowest (5.37 ± 1.17); Health literacy was positively correlated with quality of life (*r* = 0.409, *p* < 0.001) and out-of-hospital adherence (*r* = 0.223, *p* < 0.001). Adherence was also positively correlated with quality of life (*r* = 0.461, *p* < 0.001). The standardized indirect effect through adherence was 0.087 (bootstrap 95% CI: 0.048–0.134), corresponding to approximately 21.3% of the total association in the specified model.

**Conclusion:**

These cross-sectional findings carry public-health-relevant implications for older adult stroke survivors, a fast-growing vulnerable subgroup amid China’s population aging. Population-level targeted strategies to improve health literacy and post-discharge adherence may advance healthy aging by mitigating stroke-related health disparities among older adults, particularly for socio-economically disadvantaged subgroups including those with low educational attainment and rural residence. Further implementation research is required to translate these observational associations into community-tailored preventive interventions for aging populations.

## Introduction

Stroke is an acute cerebrovascular condition resulting from the abrupt obstruction or rupture of a blood vessel, which negatively impacts brain tissue (1). The most recent Global Burden of Disease (GBD) 2021 report indicates that stroke is the second leading cause of death globally, accounting for approximately 7 million fatalities, and the third leading cause of death and disability combined, measured in disability-adjusted life-years lost (DALYs), exceeding 160 million DALYs among non-communicable diseases (NCDs) (2, 3). The global financial burden of stroke is projected to surpass US$890 billion annually (0.66% of global GDP) and is anticipated to nearly treble by 2050 (4, 5). The consequences of stroke are especially obvious in densely populated China (5, 6). In 2019, China documented around 3.9 million new stroke cases and a total of 28.8 million cases, leading to 2.2 million fatalities and 45.9 million disability-adjusted life years (6). Despite significant advancements in the treatment and outcomes of stroke, the majority of stroke survivors are unable to sustain their previous physical metrics or quality of life post-illness (7, 8). Consequently, the public health burden of stroke is substantial (9). With rapid population aging in China, stroke represents a major chronic disease public health challenge affecting large numbers of older adults, placing growing demands on post-discharge community-based self-management for healthy aging.

The acute attack phase in stroke survivors typically lasts 1 to 2 weeks, followed by a predominant stable and recovery phase. The effectiveness of rehabilitation and quality of life is significantly influenced by the patient’s self-management skills and adherence to treatment, which encompasses the extent to which individuals comply with the recommendations of healthcare providers, such as medication adherence, lifestyle modifications, and increased physical activity (10). Current expert consensus indicates that stroke survivors engaging in regular rehabilitation exercises demonstrate enhanced limb function and daily living capabilities, thereby effectively lowering the risk of subsequent strokes (11, 12). High medication adherence is significantly linked to a decreased risk of coronary heart disease, stroke, and all-cause mortality (13, 14). Adherence to medication is essential for optimal clinical outcomes (15). Moreover, adherence to lifestyle modifications is a significant factor for stroke survivors. The identified risk factors may contribute to the exacerbation of symptoms and the recurrence of the disease. Nonetheless, substantial deficiencies exist in reality. Research indicates that in stroke management, adherence to rehabilitation treatment is below 50%, with a significant number of patients discontinuing the treatment plan after six months (15–18). Approximately 40 to 50% of patients do not adhere to prescribed cardiovascular medication regimens for managing their condition (19). Adherence serves as a crucial factor in the rehabilitation outcomes for stroke survivors and represents a central focus in the overall management of stroke care.

The World Health Organization (WHO) defines quality of life as the subjective assessment of life status in relation to personal goals, expectations, standards, and concerns across various cultural contexts (20). Quality of life is a comprehensive and multifaceted concept encompassing physical health, psychological well-being, autonomy, social connections, personal beliefs, spiritual support, and the living environment (21, 22). Prior research indicates that the quality of life in stroke survivors is influenced by various factors, including sociodemographic characteristics (e.g., age, education level, marital status), disease severity (e.g., hemiplegic side, complications), and the extent of motor function recovery (23–31). The recovery of motor function is identified as the most significant predictor of quality of life in chronic patients (32, 33). Insufficient adherence impedes the recovery of patients’ motor function, directly obstructs the rehabilitation process, and subsequently diminishes patients’ quality of life. This association arises from the transmission mechanism of adherence to rehabilitation benefits. High adherence guarantees the continuity and standardization of motor function training, thereby optimizing the effects of neurological function reconstruction and interrupting the detrimental cycle of “functional disorder → social role stripping → psychological exhaustion.” Ultimately, this leads to a comprehensive enhancement in quality of life. Improving treatment adherence among stroke survivors significantly enhances neurological recovery and daily living capabilities, thereby comprehensively elevating their quality of life.

Despite being recognized by the American Medical Association in 2008 as a “key factor in predicting medical adherence,” (34) health literacy (HL) has historically been undervalued as the central component of the “adherence-QOL cycle” in stroke therapy. Health literacy refers to an individual’s capacity to enhance and sustain optimal health, encompassing intricate skills such as reading, listening, comprehension, analysis, synthesis, and strategic planning (Joint Committee on National Health Education Standards, 1995). In conditions like stroke that necessitate prolonged therapy and swift information updates, individuals who are unable to get this information will experience “information disconnection.” Over 50% of stroke survivors possess inadequate health literacy, which directly impairs their capacity to recognize recurrence risk indicators and comprehend preventive treatment strategies, consequently diminishing rehabilitation adherence (35). Additional research have corroborated that inadequate health literacy is a primary factor impeding patients from preventing strokes and mitigating the risk of stroke recurrence (36). There exists a notable association between elevated health literacy and improved standards of healthy living (37–39). Individuals with diminished health literacy typically engage less in communication with medical personnel regarding their disease risks, whereas those with elevated health literacy are more proficient in their interactions with medical staff, resulting in an enhanced quality of life.

However, the precise mechanism—specifically, the extent to which out-of-hospital adherence mediates the relationship between health literacy and quality of life in stroke survivors—remains inadequately quantified and empirically validated in the Chinese context. Therefore, this study aims to: (1) Describe the levels of health literacy, adherence, and quality of life; (2) Examine the relationships among these variables; and (3) Test the hypothesis that out-of-hospital adherence will statistically partially mediate the cross-sectional association between health literacy and quality of life.

### Hypotheses

1. Health literacy will be positively associated with quality of life in stroke survivors.
2. Out-of-hospital adherence will be positively associated with both health literacy and quality of life.
3. Out-of-hospital adherence will statistically mediate part of the association between health literacy and quality of life in the prespecified cross-sectional model.

## Methods

### The study

#### Aim

This study aimed to examine associations among health literacy, out-of-hospital adherence, and quality of life, including the indirect association through adherence in a cross-sectional mediation model.

#### Theoretical framework

The existing theoretical frameworks regarding the correlation between health literacy and health outcomes are currently ambiguous. Consequently, there are clear discrepancies in the definitions and assessment methodologies among different studies. Currently, numerous distinct explanations and constructs of theoretical models or frameworks exist regarding the relationship between health literacy and health outcomes (40). It is noted that a comprehensive theoretical framework to elucidate health literacy is lacking (41, 42). This study primarily utilizes the established health literacy framework to develop its theoretical framework, encompassing: Baker’s 2006 definition of health literacy, the model depicting the relationship between health literacy and health outcomes, and the health literacy skills (HLS) model. Both models present a comprehensive framework describing how health literacy may be associated with health outcomes via mediating variables. Consequently, we employ these two frameworks as the foundation of this investigation. Within this perspective, adherence may be considered an intermediary or procedural variable, while QoL represents the ultimate outcome (43–48). Figure 1 presents a comprehensive theoretical framework illustrating the potential determinants of health outcomes in stroke survivors. Within this broader context, the current study empirically tests the specific pathway highlighted in the model (solid arrows): the mediating role of out-of-hospital adherence in the relationship between health literacy and quality of life. Other elements of the framework, such as social support, are acknowledged as important contextual factors but are not the primary focus of this analysis.

**Figure 1:**
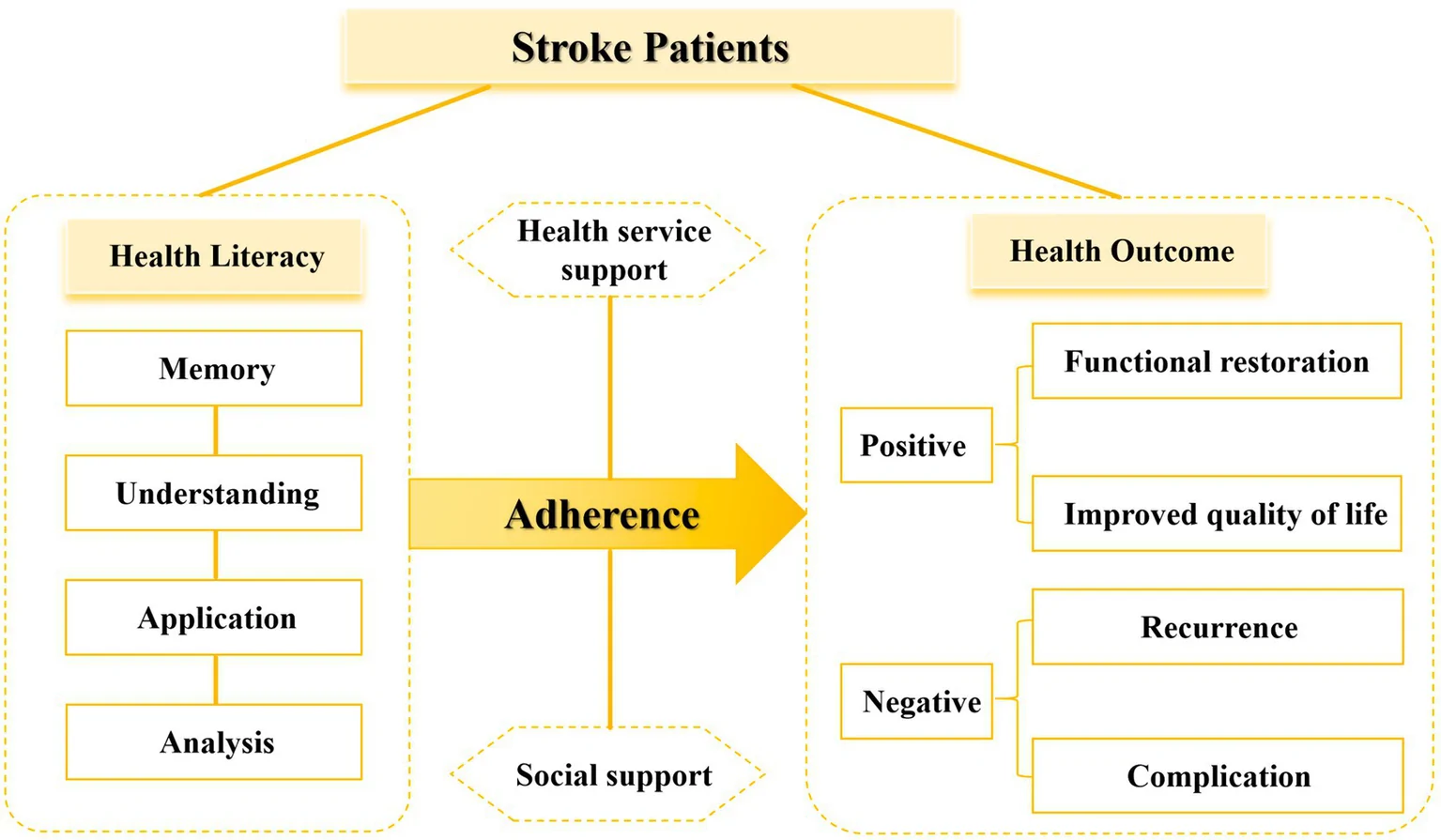
Theoretical framework.

#### Design

This study applied a cross-sectional design.

#### Study population and procedures

Participants were recruited through convenience sampling from Shanghai Hospital of Traditional Chinese Medicine, Yueyang Hospital of Integrated Traditional Chinese and Western Medicine affiliated with Shanghai University of Traditional Chinese Medicine, Shuguang Hospital affiliated with Shanghai University of Traditional Chinese Medicine, and Longhua Hospital affiliated with Shanghai University of Traditional Chinese Medicine in Shanghai between August 2023 and February 2024. This was a non-probability sample; no population-based sampling frame was used. Recruitment took place in the outpatient rehabilitation departments, neurology follow-up clinics and ward follow-up visiting areas of the four participating traditional Chinese medicine hospitals. Eligible patients were approached by trained research nurses and research team members during routine outpatient follow-up consultations and post-discharge ward follow-up visits.

*A priori* power analysis was conducted using G*Power 3.1. For a multiple linear regression with 10 predictors, detecting a small effect size (f^2^ = 0.05) with 95% power at α = 0.05 required a sample size of 473. Accounting for a 10% attrition rate, we aimed to recruit 520 participants.

*Inclusion criteria*: (1) history of transient ischemic attack (TIA) or stroke, (2)1 to 6 months post-illness and stable, (3) proficiency in language and effective communication, and (4) willingness to participate in this research.

*Exclusion criteria*: (1) severe complications, cognitive impairment, or mental disorders; (2) visual or mental impairments that hindered their ability to complete the assessment; (3) employment in medical, nursing, or other healthcare-related fields; and (4) voluntary withdrawal from the study for any reason.

## Measurements

### Demographic characteristics

The evaluated demographic parameters were gender, age, monthly income, among others, totaling 13 elements.

### Health literacy assessment

Health literacy in this study is defined as stroke survivors’ ability to remember, comprehend, apply and analyze disease-related health information to guide self-management behaviors, consistent with the conceptual framework proposed by Baker and the Health Literacy Skills Model; this construct reflects cognitive capacity rather than pure medical knowledge. We selected the simplified Chinese version of the Chronic Disease Health-Literacy Questionnaire (CHLCC) for stroke patients, an instrument culturally adapted for Chinese chronic-disease populations with well-established psychometric properties specifically among stroke survivors. Its theoretically-derived measurement model consists of four sub-dimensions: memory, comprehension, application, and analysis, with a maximum total score of 48 points. Memory is scored based on correct responses in Part 1 (1-point for each correct answer, maximum 12 points); comprehension is summed from correct answers to items B4, B8, B11, and B12 (3-points per correct answer, maximum 12 points); application is calculated from correct responses to B2, B5, B9, and B10 (3-points per correct answer, maximum 12 points); and analysis is computed from correct answers to B1, B3, B6, and B7 (3-points per correct answer, maximum 12 points). A total score ≥ 36 indicates “adequate health literacy”, while scores below 36 indicate insufficient health literacy. The questionnaire has test–retest reliability above 0.8 and a KMO value of 0.914 from prior validation work; in our present sample, the scale showed high internal consistency with a Cronbach’s α of 0.91. For our mediation model, the total scale score was adopted as the primary independent variable, whereas sub-dimension scores were used only for exploratory correlation analyses (49).

### Out-of-hospital adherence questionnaire for stroke patients (OHAQ-SP) assessment

Out-of-hospital adherence, our mediating construct, refers to the degree to which stroke survivors follow post-discharge clinical recommendations across medication taking, self-monitoring, home-based rehabilitation and lifestyle modification after hospital discharge, rather than medication adherence alone. To measure this construct, we used the Out-of-Hospital Adherence Questionnaire for Stroke Patients (OHAQ-SP), a newly developed instrument created by Sun et al. (50) to fill the gap in evaluating community-based adherence behaviors among stroke survivors. Grounded in the Harmed Behavior theory, this scale follows a four-factor measurement model encompassing medication adherence, self-monitoring adherence, rehabilitation adherence, and life-behavior-change adherence; its four-factor confirmatory factor model has been previously validated in stroke populations with satisfactory composite reliability and average variance extracted values, supporting robust construct validity. Higher total scores reflect greater self-reported adherence, though these are subjective self-reports and not independently verified objective measures of risk-factor control. For our primary analysis in PROCESS Model 4, we adopted the overall total adherence score as the mediating variable to capture this broad post-discharge behavioral construct. This decision was theoretically driven by our a-priori hypothesis focusing on the global behavioral pathway connecting health literacy to quality of life, instead of examining dimension-specific separate mediation effects; sub-dimension scores were reserved only for exploratory bivariate correlation analyses. Acceptable internal consistency of OHAQ-SP was observed within our study sample (Cronbach’s α = 0.902).

### Stroke-specific quality of life (SS-QOL) assessment

Stroke-specific quality of life (SS-QOL) is a multi-dimensional patient-reported outcome construct capturing physical, psychological, social and functional well-being among stroke survivors rather than being limited to physical function alone. In the present study, we adopted the Chinese-adapted SS-QOL scale modified by Wang Yilong et al. Its measurement model consists of 12 inter-related dimensions, including physical fitness (3 items), family role (3 items), language (5 items), activity ability (6 items), emotion (5 items), personality (3 items), self-care ability (5 items), social role (5 items), thinking (3 items), upper-limb function (5 items), vision (3 items), and work (3 items) (51). This scale adopts a 5-point Likert response format ranging from “completely like this (completely difficult)” to “completely not like this (no difficulty at all)”, yielding total scores ranging from 49 to 245, where higher total scores indicate better stroke-specific quality of life. For our mediation model, the overall SS-QOL total score was designated as the dependent variable Y to represent global stroke-related quality-of-life status, and acceptable internal consistency was observed in our sample (Cronbach’s α = 0.76).

### Statistical analysis

The study utilized SPSS 22.0 (SPSS Inc., IBM Corp., Chicago, IL, USA) for all statistical analyses. Descriptive statistics were employed to characterize variable distributions: continuous variables were expressed as mean ± standard deviation or median (interquartile range), while categorical variables were described using frequencies and percentages. Pearson correlation coefficients were calculated to examine associations among health literacy, out-of-hospital adherence, and quality of life. To test the hypothesized mediation model, we employed Model 4 of the PROCESS macro (Version 4.2) with 5,000 bias-corrected bootstrap samples. The analysis assessed both the direct statistical association of health literacy with quality of life and the indirect statistical association operating via out-of-hospital adherence in this cross-sectional dataset. Analyses of factors associated with health literacy were conducted separately from the mediation analysis. In the mediation model, health literacy was specified as X, out-of-hospital adherence as M, and quality of life as Y.

Key demographic and clinical variables that were significant in univariate analyses (*p* < 0.05) or clinically relevant were included as covariates in all regression models. Specifically, the covariates entered into the PROCESS Model 4 mediation model included age, education level, monthly household income, residence type, stroke recurrence status, and sequelae status. Important clinical variables including stroke severity, aphasia, depression, functional disability, social support, and caregiver burden were not collected in the present study and therefore were not adjusted for in the mediation models. Mediation effects were considered statistically significant if the 95% confidence intervals did not include zero. For all analyses, statistical significance was set at *p* < 0.05. Prior to analyses, the normality of continuous variables was assessed using the Shapiro–Wilk test and visual inspection of Q-Q plots. Bootstrap confidence intervals were used because the sampling distribution of the indirect effect may be asymmetric. Bootstrapping does not resolve confounding, model misspecification, or violations of the assumed functional relationships. Assumptions of linearity and homoscedasticity were visually evaluated via residual plots, and no severe model violations were detected.

### Ethical considerations

All personally identifiable information in the study was encrypted to safeguard the participants. The scales obtained in this study were anonymous, and all data was rigorously protected. The study was approved by the Ethics Committee of Shanghai Municipal Hospital of Traditional Chinese Medicine (approval no. 2018SHL-KY-16) and was conducted in accordance with the Declaration of Helsinki. Written informed consent was obtained from all participants before data collection. For participants with mild communication difficulties caused by stroke, trained research staff or their family members provided neutral, non-leading assistance during questionnaire completion. No suggestive or leading prompts were given; participants retained full control over all survey responses. Individuals with severe cognitive impairment were excluded per study eligibility criteria.

## Results

### Descriptive statistics

Of the 520 questionnaires received, 11 were excluded because of missing data on key study variables, leaving 509 complete questionnaires for analysis. The proportion of complete questionnaires was 97.88%. For the remaining dataset, list-wise deletion was applied to handle sporadic missing values in non-key secondary variables. No systematic pattern of missingness was identified for study variables. The mean health literacy score was 31.40 ± 6.78, below the instrument’s 36-point threshold for adequate health literacy (Table 1).

**Table 1:** Scores of health literacy dimensions among stroke survivors (*n* = 509).

Dimensions	Score	Complete score percentage^1^
Recall	10.33 ± 1.74	86.05%
Comprehend	8.71 ± 1.21	72.50%
Application	6.99 ± 1.48	58.20%
Analyze	5.37 ± 1.17	44.75%
Total score^2^	31.40 ± 6.78	

Data are presented as mean ± standard deviation (SD). ^1^The complete-score percentage represents the proportion of maximum attainable score for each dimension, calculated as (mean score/maximum possible score) × 100%. ^2^Maximum possible score for each sub-dimension: Recall = 12 points, Comprehend = 12 points, Application = 12 points, Analyze = 12 points; overall maximum total score = 48 points.

### Demographic and clinical factors associated with health literacy

The analysis of intergroup differences in health literacy scores indicated that single-factor variables, including gender, marital status, occupational status, chronic disease status, type of medical insurance, and living conditions, were not associated with statistically significant differences in health literacy scores (*p* > 0.05). Health literacy scores differed significantly across categories of education, residence, stroke history, sequelae status, and income (*p* < 0.05). *Post hoc* multiple comparisons utilizing the Tukey method revealed a positive correlation between educational attainment and health literacy scores, which increased significantly with higher education levels. Urban residents exhibited significantly higher scores than their rural counterparts (*F* = 4.76, *p* < 0.01). Additionally, non-first-episode patients demonstrated significantly higher health literacy scores compared to first-episode patients (*t* = −2.442, *p* < 0.05). Patients without sequelae scored significantly higher than those with sequelae (*F* = 4.960, *p* < 0.01). Furthermore, individuals with an annual income exceeding 20,000 yuan had significantly higher scores than those earning less than 1,000 yuan (*F* = 2.838, *p* < 0.05) (Table 2).

**Table 2:** Univariate analysis of factors associated with health-literacy scores (*n* = 509, Mean ± SD).

Demographic characteristics	*n* (%)	Health literacy assessment score ( $\bar{x}$ ±S)^2^	F/*t*^1^	*p*
Gender				
Male	276 (54.22%)	31.17 ± 6.79	−0.74	0.46
Female	233 (45.78%)	31.62 ± 6.77		
Age				
<50	25 (4.92%)	30.11 ± 6.99	0.654	0.55
51 ~ 60	72 (14.15%)	31.05 ± 6.23		
61 ~ 69	285 (55.99%)	31.29 ± 6.18		
≥70	127 (24.95%)	30.53 ± 6.14		
Education				
Illiteracy	181 (35.56%)	30.34 ± 6.89	4.30	0.005
Elementary or Middle school	125 (24.56%)	31.14 ± 6.18		
Senior secondary school	161 (31.63%)	32.01 ± 6.85^*^		
University or above	42 (8.25%)	34.12 ± 6.89^**^		
Marital status				
Married	300 (58.94%)	31.69 ± 7.06	0.55	0.65
Divorce	44 (8.64%)	30.77 ± 5.67		
Widowed	115 (22.59%)	30.88 ± 6.45		
Unmarried	50 (9.82%)	31.20 ± 6.71		
Occupation				
Incumbency	292 (57.37%)	31.36 ± 6.82	1.32	0.27
Unemployment	43 (8.45%)	32.23 ± 5.69		
Retired	134 (26.33%)	30.71 ± 6.93		
Others	40 (7.86%)	32.88 ± 6.94		
Residency				
Countryside	320 (62.87%)	30.80 ± 6.98	4.76	0.009
Towns	125 (24.56%)	31.75 ± 5.95^*^		
Municipalities	64 (12.57%)	33.56 ± 6.86^**^		
Combined chronic diseases				
One	244 (47.94%)	31.37 ± 6.70	1.30	0.27
Two	208 (40.86%)	31.74 ± 6.25		
Three or above	57 (11.20%)	30.11 ± 7.58		
Initial stroke				
Yes	182 (35.76%)	30.40 ± 6.46	−2.442	0.015
No (Recurrent)	327 (64.24%)	31.93 ± 6.90		
Type of medical insurance				
Urban residents	129 (25.34%)	31.14 ± 6.99	0.112	0.89
New Rural Cooperative Medical Insurance	178 (34.97%)	31.43 ± 7.26		
Others	202 (39.69%)	31.49 ± 6.20		
Residence				
Solitary	184 (36.15%)	31.24 ± 7.19	−0.351	0.73
Co-habitation	325 (63.85%)	31.46 ± 6.54		
Sequelae				
Yes	170 (33.40%)	30.10 ± 6.70	4.960	0.007
No	243 (47.74%)	32.21 ± 6.92^**^		
Yes, but recovered	96 (18.86%)	31.54 ± 6.23^*^		
Incomes (RMB/month)^3^				
<1,000	59 (11.59%)	27.65 ± 6.76	2.838	0.01
1,001–3,000	86 (16.90%)	31.74 ± 6.88^*^		
3,001–6,000	136 (26.72%)	31.57 ± 6.57^*^		
6,001–10,000	118 (23.18%)	31.03 ± 5.37^*^		
10,001–20,000	74 (14.54%)	31.00 ± 8.82^*^		
>20,000	31 (6.09%)	33.59 ± 5.94^**^		
Have no idea	5 (0.98%)	30.80 ± 7.73		

Data are presented as mean ± standard deviation (SD). ^1^Statistical tests used: Independent samples *t*-test was used for binary variables (e.g., Gender); One-way analysis of variance (ANOVA) was used for multi-category variables (e.g., Education). The F-statistic is reported for ANOVA, and the *t*-statistic for *t*-tests. ^2^*indicate significant differences compared to the reference group (the first category listed for each variable) using post-hoc Dunnett’s test. ^3^RMB: Chinese Yuan. Income is measured per month. *p < 0.05, **p < 0.01.

A subsequent hierarchical regression analysis was conducted. The findings indicated that the explanatory capacity of model 1 (including just control variables) for health literacy was 9.2% (R^2^ = 0.092, *F* = 3.872). Incorporating the patient’s out-of-hospital adherence and quality of life variables significantly enhanced the explanatory power of model 2 to 24.4% (ΔR^2^ = 0.152, *p* < 0.001; *F* = 10.608). Among the control variables, education level exhibited a step effect (college and above *β* = 0.130** > high school/college *β* = 0.104), economic capital was significant (high-income group *β* = 0.189**), and the absence of sequelae was a crucial protective factor (*β* = 0.129**).

Higher education, higher income, and absence of sequelae were associated with higher health literacy scores in the reported model. Education level demonstrated a step effect: respondents with college and above education had (*β* = 0.130, *p* < 0.01), while those with high school/college education had (*β* = 0.104). The high-income group showed (*β* = 0.189, *p* < 0.01), and absence of sequelae acted as a critical protective factor with (*β* = 0.129, *p* < 0.01). The variance inflation factors (VIFs) for all predictors across models were below 3, indicating that multicollinearity was not a critical issue in the interpretation of the regression coefficients. These findings identify socioeconomic and clinical correlates of health literacy but do not establish the mechanisms underlying the observed differences (Table 3).

**Table 3:** Hierarchical regression analysis of factors associated with health-literacy scores.

Dependent variable	Health literacy			
	Model 1		Model 2	
	Beta	VIF^1^	Beta	VIF^1^
Independent variable				
Out-of-hospital adherence			0.052	1.298
Quality of life			0.368***	1.299
Control variables				
Education^2^				
Elementary or Middle school (dummy variable1)	0.043	1.283	0.057	1.286
Senior secondary school (dummy variable 2)	0.110*	1.322	0.104*	1.322
University or above (dummy variable 3)	0.120**	1.164	0.130**	1.165
Residency^2^				
Towns (dummy variable 1)	0.056	1.055	0.055	1.056
Municipalities (dummy variable 2)	0.101*	1.096	0.100*	1.096
Initial stroke	0.094*	1.029	0.075	1.033
Sequelae^2^				
No (dummy variable 1)	0.147**	1.281	0.129**	1.284
Yes, but recovered (dummy variable 2)	0.073	1.280	0.053	1.284
Incomes^2^				
1,000–3,000	0.185*	2.837	0.133*	2.865
3,001–6,000	0.195*	3.531	0.165*	3.547
6,001–10,000	0.148	3.323	0.131	3.328
10,001–20,000	0.122	2.643	0.072	2.659
>20,000	0.240***	2.355	0.189**	2.372
R^2 3^	0.092		0.244	
ΔR^2 3^	0.092		0.152	
F	3.872***		10.608***	

Beta values are standardized regression coefficients. ^1^VIF, Variance Inflation Factor, used to detect multicollinearity; values < 10 indicate acceptable multicollinearity. ^2^Dummy variables were created for categorical variables with the following reference categories: Education - Illiteracy; Residency - Countryside; Sequelae - Yes (with sequelae); Incomes - < 1,000 RMB/month. ^3^R^2^, Coefficient of determination; ΔR^2^, Change in R^2^ between nested models. *p < 0.05, **p < 0.01, ***p < 0.001.

### Study on the correlation among health literacy, out-of-hospital adherence, and quality of life

Bivariate Pearson correlation analysis (*n* = 509) revealed distinct patterns of association among health literacy, out-of-hospital adherence, and quality of life. According to Cohen’s criteria for effect sizes—where |*r*| ≥ 0.10, 0.30, and 0.50 indicate small, medium, and large effects, respectively—health literacy demonstrated a weak positive correlation with out-of-hospital adherence (*r* = 0.223, *p* < 0.001) and a moderate positive correlation with quality of life (*r* = 0.409, *p* < 0.001). In contrast, out-of-hospital adherence exhibited a stronger correlation with quality of life (*r* = 0.461, *p* < 0.001), approaching a large effect size. Among the bivariate correlations examined, adherence had the largest observed correlation with quality of life. Both the adherence–quality-of-life correlation and the health literacy-quality of life correlation were moderate under the stated conventions (Table 4).

**Table 4:** Pearson correlation coefficients among health literacy, out-of-hospital adherence, and quality of life.

Variables	Health literacy^1^	Out-of-hospital adherence^1^	Quality of life^1^
Health literacy			
Out-of-hospital adherence	0.223***		
Quality of life	0.409***	0.461***	
Mean±SD^2^	31.379 ± 6.775	90.018 ± 11.515	177.902 ± 15.105

Data are presented as correlation coefficients (Pearson’s *r*) and mean±SD. ^1^All correlation coefficients were statistically significant at *p* < 0.001 level. ^2^The bottom row displays mean ± standard deviation values for each variable. *p < 0.05, **p < 0.01, ***p < 0.001.

An extensive analysis of the correlations among questionnaire domains of the multi-dimensional questionnaire utilizing Pearson bivariate correlation revealed a significant positive correlation between quality of life and all sub-dimensions of out-of-hospital adherence (all *P* < 0.01). Rehabilitation adherence exhibited the highest correlation strength (*r* = 0.370), whereas self-monitoring adherence demonstrated the weakest correlation (*r* = 0.243). Additionally, all four dimensions of health literacy were significantly positively correlated with quality of life (all *P* < 0.01), with analytical ability showing the strongest correlation (*r* = 0.322) and the memory dimension showing the smallest observed correlation (*r* = 0.164). The correlations between adherence and health literacy domains revealed that the memory dimension exhibited no significant association with the adherence sub-dimensions (all *p* > 0.05), however the application dimension demonstrated a significant positive correlation with all adherence sub-dimensions (all *p* < 0.05) (Table 5).

**Table 5:** Analysis of correlations among the dimensions of the questionnaire.

Variables^2^	1	2	3	4	5	6	7	8	9
Quality of life	1								
Medication adherence	0.305**	1							
Self-monitoring adherence	0.243**	0.511**	1						
Rehabilitation adherence	0.370**	0.125**	0.293**	1					
Life behaviors change adherence	0.287**	0.031	0.098*	0.391**	1				
Memory_HL^1^	0.164**	−0.012	−0.043	0.040	0.067	1			
Comprehension_HL^1^	0.192**	0.084	0.063	0.080	0.036	0.244**	1		
Application_HL^1^	0.305**	0.171**	0.119**	0.165**	0.099*	0.037	0.101*	1	
Analysis_HL^1^	0.322**	0.175**	0.112*	0.160**	0.076*	0.102*	0.290**	0.281**	1

Correlation matrix displaying Pearson correlation coefficients among questionnaire dimensions. ^1^HL, Health Literacy; Memory_HL, Memory dimension of health literacy; Comprehension_HL, Comprehension dimension of health literacy; Application_HL, Application dimension of health literacy; Analysis_HL, Analysis dimension of health literacy. ^2^All correlations are two-tailed. The matrix shows intercorrelations among quality of life, medication adherence domains, and health literacy dimensions. *p < 0.05, **p < 0.01.

### The mediating role of out-of-hospital adherence

A three-step methodology for assessing mediation effects was utilized. The study encompasses independent variable X, dependent variable Y, and mediating variable M (Figure 2).

**Figure 2:**
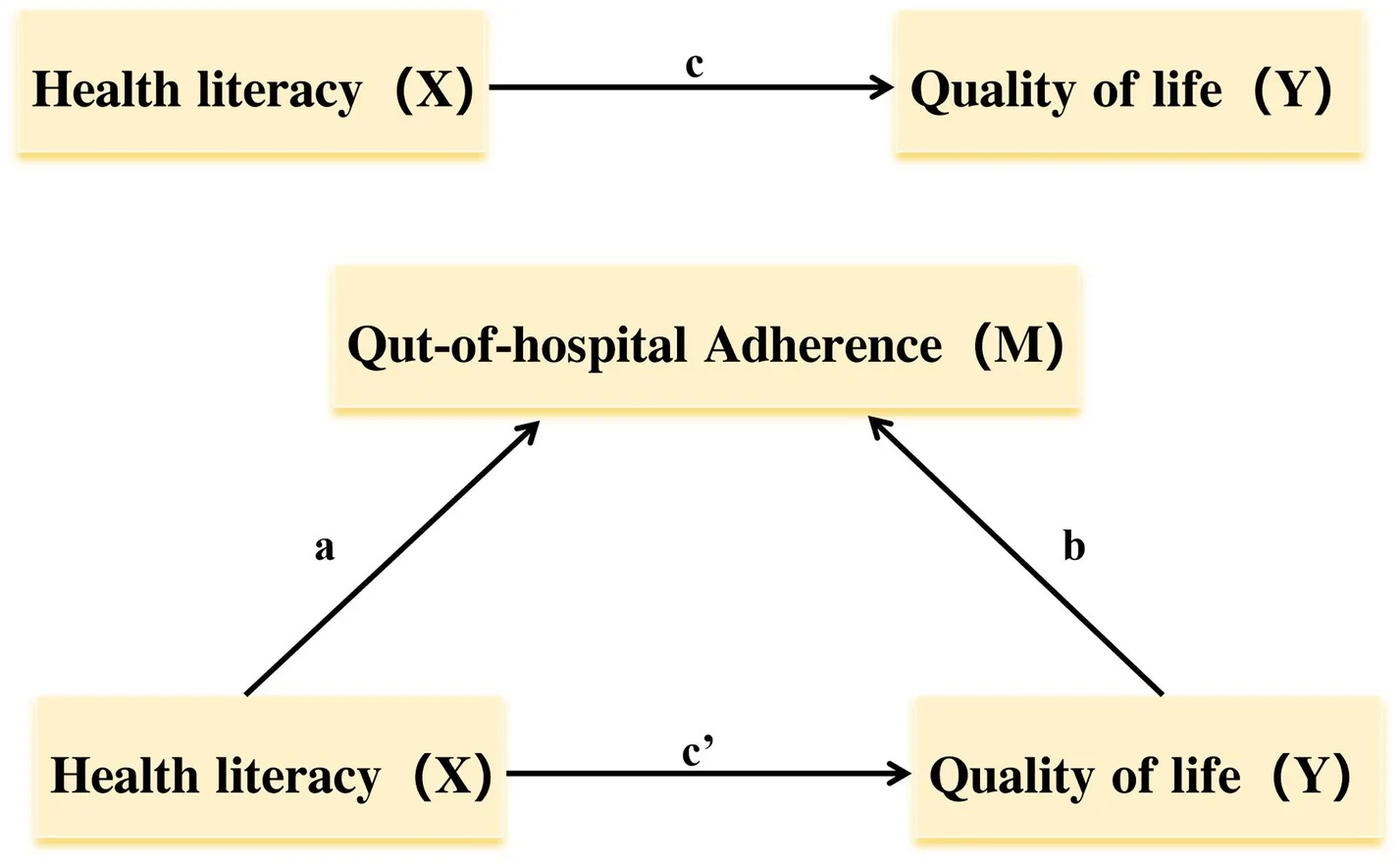
Statistical partial mediation model. Coefficients c, a, b, c’ represent paths in the statistical mediation model derived from cross-sectional data. Coefficient c = total statistical association of X on Y; a = statistical association of X on M; b = statistical association of M on Y adjusted for X; c’ = direct statistical association of X on Y adjusted for M. The product ab corresponds to the indirect statistical association. Total statistical association: c = c’ + ab. These statistical path coefficients describe observed cross-sectional correlations and do not prove causal temporal relationships.

The mediation analysis, utilizing hierarchical regression, revealed that health literacy was associated with quality of life through both direct and indirect pathways. In Model 1, health literacy demonstrated a substantial direct positive association on quality of life (*β* = 0.409, *p* < 0.001), accounting for 16.7% of its variance (R^2^ = 0.167, *F* = 101.789**); Model 2 indicates that health literacy was significantly associated with higher out-of-hospital adherence (*β* = 0.223, *p* < 0.001), albeit with limited explanatory power (R^2^ = 0.050); Model 3 demonstrates that when health literacy and out-of-hospital adherence are concurrently considered, the effect of health literacy is diminished yet remains highly significant (*β* = 0.322, *p* < 0.001), while out-of-hospital adherence contributes more robustly to quality of life (*β* = 0.390, *p* < 0.001). The two factors collectively accounted for 31.1% of the variance in quality of life (R^2^ = 0.311, *F* = 114.428), indicating a partial mediation model where health literacy was associated with better quality of life in part through improved adherence (indirect effect proportion = 21.3%), while maintaining 78.7% of the direct effect (Table 6).

**Table 6:** Evaluation of out-of-hospital adherence as a mediating construct between health literacy and quality of life.

Dependent variable	Quality of life (Model 1)^2,3^	Out-of-hospital adherence (Model 2)^2,3^	Quality of life (Model 3)^2,3^
Independent variable			
Health literacy	0.409***	0.223***	0.322***
Mediating variables			
Out-of-hospital adherence			0.390***
R^2 1^	0.167	0.050	0.311
F	101.789***	26.581***	114.428***

Standardized regression coefficients (*β*) are reported for each path in the mediation analysis. ^1^R^2^, Coefficient of determination, representing the proportion of variance explained by the model. ^2^Models are based on cross-sectional linear regression to evaluate the statistical mediating association of out-of-hospital adherence; these do not confirm causal pathways. ^3^Model 1: Total association of health literacy with quality of life (c); Model 2: Association of health literacy with adherence (a); Model 3: Conditional associations of health literacy (c′) and adherence (b) with quality of life. *p < 0.05, **p < 0.01, ***p < 0.001.

The subsequent analysis of the mediation effect revealed a direct effect value of 0.322 (95% CI: 0.224–0.429, *p* < 0.001), constituting 78.73% of the total effect. The indirect effect, mediated by out-of-hospital adherence, had an effect value of 0.087 (95% CI: 0.048–0.134, Z = 2.811, *p* = 0.005), representing 21.27% of the total effect. The overall effect was 0.409 (95% CI: 0.309–0.505). In this cross-sectional model, health literacy was predominantly statistically associated with better quality of life via direct pathways (78.7%), which may reflect cognitive-related resources among participants. Meanwhile, out-of-hospital adherence contributed 21.3% to the total statistical indirect association in this model. These correlational findings suggest potential directions for future intervention research: targeting health literacy (especially analytical and application dimensions) and out-of-hospital adherence may hold promise for improving post-stroke quality of life, though such hypothesized benefits require formal verification in intervention trials (Table 7).

**Table 7:** Analysis of mediation effects.

Intermediate route	Effect	Magnitude of effect	Bootstrap95%CI ^1^		Sobel test ^3^	Proportional value ^2^
			Lower limit	Upper limit		
Health literacy ->Out-of-hospital adherence ->Quality of life	Direct statistical effect	0.322	0.224	0.429		78.73%
	Indirect statistical effect^3^	0.223*0.390 = 0.087	0.048	0.134	2.811 *p* = 0.005	21.27%
	Total statistical effect	0.322 + 0.087 = 0.409	0.309	0.505		

Mediation analysis was conducted using bootstrapping procedures with 5,000 resamples. ^1^CI, Confidence Interval. Bootstrap 95% CIs that do not include zero indicate statistically significant statistical associations. ^2^The proportional value represents the proportion of the total statistical effect accounted for by the indirect statistical effect, derived from cross-sectional data and does not reflect causal contribution. ^3^The indirect statistical effect was calculated as the product of path coefficients (0.223 × 0.390 = 0.087). The Sobel test was used to test the significance of this statistical mediation association.

## Discussion

This study describes observed associations among health literacy, out-of-hospital adherence, and quality of life among stroke survivors based on cross-sectional empirical findings. The overall health literacy score of 31.43 ± 6.78, which is below the 36-point threshold, suggests suboptimal health literacy within this sample, with notable variations across subdomains. Patients exhibited a relative strength in the memory dimension, with a mean score of 10.33 ± 1.74, especially for concrete terms like “blood pressure” and “exercise.” However, they encountered difficulties with abstract medical terminology, such as “spasticity.” This is consistent with existing literature suggesting that in groups with limited health literacy, the ability to recognize basic health information typically surpasses their interactive or critical literacy skills (52–54).

The comprehension score of 8.71 ± 1.21 suggests that patients in this sample possessed a reasonable understanding of follow-up protocols and rehabilitation principles, consistent with proactive involvement in care continuity. This finding contrasts with the observation by Morris et al. (55) that low literacy is associated with decreased follow-up attendance and information-seeking behavior. This discrepancy may be explained by stroke-specific motivational factors, including fear of recurrence or the severity of disability, which could promote engagement despite limitations in literacy.

The application (6.99 ± 1.48) and analysis (5.37 ± 1.17) dimensions showed the lowest performance. Challenges within the application dimension were evident mainly in medication management and lifestyle modifications, reflecting self-reported difficulties among participants in translating theoretical knowledge into sustained self-care practices. This mismatch may be related to cognitive burdens associated with post-stroke polypharmacy and intricate rehabilitation protocols. Poor performance on analysis-related tasks was notable for contextual reasoning requirements, such as interpreting medication interactions and individualizing general clinical advice, reflecting self-reported weaknesses in critical health-related decision-making. These observed deficits align with the well-documented “knowledge–behavior gap,” in which knowledge recall and comprehension do not reliably translate into corresponding health behaviors. This pattern points not only to informational gaps among participants but also potential shortfalls within care delivery systems: standardized educational resources may insufficiently accommodate stroke-related cognitive barriers, and patient education often prioritizes knowledge transfer rather than practical behavioral support. These observed gaps in health literacy sub-dimensions carry particular public-health relevance for older-adult stroke survivors amid China’s population aging. Many community-dwelling older adults face extra barriers when translating medical knowledge into self-care behaviors, given age-related cognitive changes, limited digital access, and uneven exposure to age-friendly health educational resources.

Multivariable analysis showed that education (*β* = 0.32, *p* < 0.001), residence type, stroke recurrence history, and income were correlated with health-literacy scores. Participants with university-level education scored higher than those with primary-level education. This educational gradient may relate to better information-processing capacity, greater use of digital resources, and more effective communication with healthcare providers, although these pathways cannot be verified from cross-sectional data. Observed urban–rural differences may reflect uneven access for urban residents to concentrated healthcare resources, specialized rehabilitation facilities, and digital-health infrastructure; rural participants may face geographic barriers, cultural challenges to lifestyle adjustment, and limited tailored stroke educational materials. Participants with recurrent stroke scored higher than first-stroke participants. This pattern may reflect experiential “health capital” from prior clinical encounters that reduces information avoidance, but causal inference is limited. Lower-income participants appeared to face financial constraints on health-related resources and opportunity costs, such as lost income during medical visits, reducing engagement with health information; longitudinal investigation is warranted. These socioeconomic gradients reflect broader health inequities among vulnerable older adult stroke survivors. Older adults with low education, rural residency, and low household income are high-priority groups for public-health-led chronic-disease management. Hospital-based clinical education alone cannot fully address these multilayered disadvantages, aligning with this section’s call for research on underserved aging populations and healthy-aging-oriented preventive strategies. In our sample, most participants were aged 61–69 years, followed by those aged ≥70 years. Although univariate analysis showed no statistically significant age-stratified differences in health-literacy scores, the advanced old-age subgroup (≥70 years) trended toward lower performance. This underscores the importance of age-tailored public-health strategies, as physiological and cognitive changes among the oldest old may compound barriers to processing stroke-related health information, even after adjusting for education and socioeconomic status.

Our finding that 78.7% of the total statistical association between health literacy and quality of life represented the direct path aligns with prior literature highlighting the relevance of cognitive-related factors for stroke survivor well-being (31, 39). It should be emphasized that this is a statistical partial mediation derived from cross-sectional data rather than a confirmed causal mechanism. The proportion of the total association accounted for by the indirect path via out-of-hospital adherence was 21.27%, lower than estimates reported among cardiac disease populations (30–40%) (38), pointing toward potential condition-specific patterns. In the present study, out-of-hospital adherence was measured using the OHAQ-SP scale covering four domains: medication adherence, self-monitoring adherence, home-based rehabilitation adherence, and lifestyle-modification adherence. Health literacy was operationalized using the Chinese Chronic-Disease Health-Literacy Questionnaire evaluating memory, comprehension, application, and analytical critical skills. Within this cross-sectional correlation structure, several plausible explanatory pathways can be hypothesized but cannot be confirmed. Notably, the present mediation analysis used the overall total score of out-of-hospital adherence as the mediating variable rather than separate sub-dimension scores. Therefore, the observed partial statistical mediation reflects global post-discharge behavioral patterns, and we cannot draw conclusions regarding mediation statistical associations for any single adherence domain (e.g., medication or rehabilitation adherence). Similarly, health literacy in this study covers memory, comprehension, application and analysis dimensions, corresponding to functional, interactive and critical health literacy skills; exploratory bivariate correlations suggested that application and analytical dimensions exhibited stronger correlations with study outcomes relative to memory dimension, which warrants further investigation in future studies.

Better information-processing capacity among participants is correlated with their ability to filter and synthesize rehabilitation knowledge from clinicians, digital sources, and peer experience, which in turn is associated with better adherence behaviors. In addition, adequate understanding of stroke may correlate with lower psychological distress through realistic symptom appraisal and recovery expectations, which in turn correlates with higher self-efficacy. Among health-literacy subdomains, analytical skills showed the strongest bivariate correlation with quality of life, consistent with the notion that critical appraisal of health-related information may support participants to adjust rehabilitation strategies according to residual physical function and engage with health services. The observed statistical partial mediation supports a hypothesized “cognitive-behavioural loop”: higher health-literacy scores correlate with higher self-reported out-of-hospital adherence, and higher adherence is further correlated with better quality-of-life outcomes. However, alternative pathways and reverse directional relationships are possible given cross-sectional measurement. Rehabilitation adherence showed the strongest bivariate correlation with quality of life in our sample; consistent engagement in rehabilitation and social interaction within group rehabilitation may correlate with better physical and psychological status. Nevertheless, this hypothesized loop may break at the transition from knowledge application and analysis to real-world behavior. Importantly, the 21.27% indirect proportion indicates that most of the association between health literacy and quality of life is not explained by out-of-hospital adherence alone. Other unmeasured or unexamined variables such as self-efficacy, depressive symptoms, social support, caregiver involvement, functional recovery, and access to rehabilitation services may serve as important alternative or parallel mediators linking health literacy to quality of life. For aging populations, this partial-mediation pattern suggests that improving post-stroke quality of life for older-adult stroke survivors cannot rely solely on single-target behavioral interventions. Multi-component public-health strategies are required that simultaneously consider cognitive capacities, social-economic contexts, as well as family-caregiving resources for older adults living with stroke.

The correlational patterns observed in this study may suggest potential directions for tailored health-service strategies, although the effectiveness of these approaches requires formal testing in intervention trials. From a section-aligned public-health perspective focused on healthy aging, these strategies should target older adult stroke survivors as a key vulnerable aging subgroup, emphasizing intervention dissemination, implementation, and clinical-community transition beyond hospital-centered care. For participants with limited health literacy, easy-to-understand educational resources such as animated decision-support tools and dialect-adapted multimedia materials deployed in community health centers may help clarify complex clinical concepts including medication safety and spasticity management. Digital health content delivered via mobile platforms holds promise to reduce geographic barriers for rural populations, though real-world effectiveness remains to be evaluated. Stratified patient education could be considered: motivational interviewing during acute stroke hospitalization may be explored for first-event patients to reduce information avoidance; peer-support groups may be evaluated for recurrent-stroke survivors to share practical self-management experience. Care-coordination models linking general practitioners and neurologists in urban settings, together with strengthened county-level medical alliances, culturally appropriate health education, and flexible clinic appointment scheduling for rural populations deserve further investigation. Financial support measures such as medication subsidies and transportation reimbursements for socio-economically disadvantaged stroke survivors may reduce cost-driven non-adherence, but whether such measures translate to better post-stroke quality of life needs empirical verification.

Participants were enrolled by convenience sampling exclusively from four traditional Chinese medicine hospitals within Shanghai. Therefore, this sample cannot be treated as representative of stroke survivors across broader China, including patients receiving care in general hospitals, primary-care institutions, patients living in rural regions outside Shanghai, or survivors from other provinces. Differences in hospital care models, discharge education content, and local rehabilitation resources may shape self-reported adherence and patient-reported quality of life outcomes; these contextual variables were not quantified in the current investigation. Even participants coded as rural residency within our sample were individuals who attended urban TCM hospitals, so they cannot represent community-dwelling rural stroke survivors who do not seek hospital-based follow-up. Furthermore, discharge health education, rehabilitation expectations and clinical management patterns may differ between TCM oriented and western medicine dominated hospitals; hence our findings may not directly generalize to stroke populations receiving care in non-TCM hospital settings. In addition, our inclusion criteria excluded individuals with cognitive impairment and severe communication barriers, meaning people with severe disability are likely underrepresented in the present sample. Future research with population-based sampling covering diverse geographic contexts and hospital systems is needed to externally validate these observed correlational patterns.

Beyond these sampling-driven threats to external validity, several additional methodological considerations should be borne in mind when interpreting these findings. All key study variables including health literacy, out-of-hospital adherence, and quality of life relied entirely on self-reported questionnaires completed at a single time-point, raising risks of common-method variance, same-source bias, recall bias, and response-style effects that may inflate the observed cross-sectional associations. Furthermore, several clinically critical stroke-related variables such as baseline stroke severity, functional disability severity, depressive symptoms, and caregiver burden were not collected in this study and thus could not be entered as adjustment covariates in the present mediation models. Residual confounding originating from these unmeasured factors cannot be ruled out, as these clinical characteristics may simultaneously shape health-literacy performance, adherence behaviors, and self-reported quality of life status.

## Conclusion

In this cross-sectional sample of Chinese stroke survivors from four Shanghai TCM hospitals, higher health-literacy correlated with better self-reported quality of life, with out-of-hospital adherence partially mediating this relationship. While supporting a plausible health-literacy-adherence-wellbeing pathway, these observational data cannot establish temporality or causality. For China’s aging population, our findings highlight modifiable public-health targets, health literacy and post-discharge adherence, to advance healthy aging and mitigate stroke-related disparities among vulnerable older adult subgroups. Longitudinal and implementation-focused public-health research in diverse older adult cohorts is required to translate these associations into real-world quality-of-life benefits.

### Limitations

This study has several key limitations. First, its cross-sectional design cannot establish temporal ordering or causal relationships. The observed partial statistical mediation only reflects correlational patterns, rather than confirming causal pathways; reverse associations and unmeasured confounding remain possible. Second, all study outcomes relied on self-reported questionnaires, which may lead to social desirability bias, common-method variance, same-source bias and recall bias that could inflate observed associations. Third, convenience sampling from four traditional Chinese medicine hospitals in Shanghai introduces selection bias. Findings may not generalize to stroke survivors in other regions, patients treated in western-medicine-oriented hospitals, community-dwelling rural patients without hospital follow-up, or individuals with severe disability and cognitive impairment. Fourth, important clinical confounders including baseline stroke severity, depressive symptoms, functional disability and caregiver burden were not collected, so residual confounding cannot be fully excluded. Additionally, measurement invariance across subgroups was not tested, and potential inter-correlation among socioeconomic variables should be noted. Lack of longitudinal data prevents exploring dynamic changes of study variables over time. These caveats should be considered when interpreting results; multi-site longitudinal and interventional studies are needed for further validation.

## Data Availability

The original contributions presented in the study are included in the article/supplementary material, further inquiries can be directed to the corresponding author.
